# Carriage Dynamics of Pneumococcal Serotypes in Naturally Colonized Infants in a Rural African Setting During the First Year of Life

**DOI:** 10.3389/fped.2020.587730

**Published:** 2021-01-08

**Authors:** Chrispin Chaguza, Madikay Senghore, Ebrima Bojang, Stephanie W. Lo, Chinelo Ebruke, Rebecca A. Gladstone, Peggy-Estelle Tientcheu, Rowan E. Bancroft, Archibald Worwui, Ebenezer Foster-Nyarko, Fatima Ceesay, Catherine Okoi, Lesley McGee, Keith P. Klugman, Robert F. Breiman, Michael R. Barer, Richard A. Adegbola, Martin Antonio, Stephen D. Bentley, Brenda A. Kwambana-Adams

**Affiliations:** ^1^Parasites and Microbes Programme, Wellcome Sanger Institute, Cambridge, United Kingdom; ^2^Darwin College, University of Cambridge, Cambridge, United Kingdom; ^3^Medical Research Council (MRC) Unit The Gambia at the London School of Hygiene and Tropical Medicine, Fajara, Gambia; ^4^Respiratory Diseases Branch, Centers for Disease Control and Prevention, Atlanta, GA, United States; ^5^Hubert Department of Global Health, Rollins School of Public Health, Emory University, Atlanta, GA, United States; ^6^Emory Global Health Institute, Emory University, Atlanta, GA, United States; ^7^Department of Infection, Immunity and Inflammation, University of Leicester, Leicester, United Kingdom; ^8^RAMBICON Immunisation & Global Health Consulting, Lekki, Nigeria; ^9^Warwick Medical School, University of Warwick, Coventry, United Kingdom; ^10^Department of Pathology, University of Cambridge, Cambridge, United Kingdom; ^11^NIHR Global Health Research Unit on Mucosal Pathogens, Division of Infection and Immunity, University College London, London, United Kingdom

**Keywords:** pneumococcus, serotype, carriage duration, acquisition, Africa, infants

## Abstract

*Streptococcus pneumoniae* (the pneumococcus) carriage precedes invasive disease and influences population-wide strain dynamics, but limited data exist on temporal carriage patterns of serotypes due to the prohibitive costs of longitudinal studies. Here, we report carriage prevalence, clearance and acquisition rates of pneumococcal serotypes sampled from newborn infants bi-weekly from weeks 1 to 27, and then bi-monthly from weeks 35 to 52 in the Gambia. We used sweep latex agglutination and whole genome sequencing to serotype the isolates. We show rapid pneumococcal acquisition with nearly 31% of the infants colonized by the end of first week after birth and quickly exceeding 95% after 2 months. Co-colonization with multiple serotypes was consistently observed in over 40% of the infants at each sampling point during the first year of life. Overall, the mean acquisition time and carriage duration regardless of serotype was 38 and 24 days, respectively, but varied considerably between serotypes comparable to observations from other regions. Our data will inform disease prevention and control measures including providing baseline data for parameterising infectious disease mathematical models including those assessing the impact of clinical interventions such as pneumococcal conjugate vaccines.

## Introduction

The pneumococcus continues to kill >320,000 children under 5 years old every year globally despite the use of highly effective serotype-specific pneumococcal conjugate vaccines (PCV) (1). Nasopharyngeal pneumococcal carriage is an essential precursor to invasive pneumococcal disease (2, 3). Furthermore, important biological and ecological events such as evolution and transmission of the pneumococcus occur during carriage (4–6). These fundamental processes influence the population-level characteristics of the strain types and serotypes including the distribution, interactions, and phenotypes such as virulence, vaccine escape, antimicrobial resistance, and immune evasion. Therefore, understanding patterns of pneumococcal carriage remains an essential component of pneumococcal epidemiology for inferences and prediction of the strain dynamics.

Mathematical models are increasingly becoming useful for investigating disease dynamics. This includes understanding transmission patterns (7), antibiotic resistance (5, 8), and impact of infant vaccination programs (9). The accuracy of these models depends on the availability of reliable data to initialize the parameters; therefore, it is crucial to conduct studies to generate such required information. Such data include carriage dynamics specifically time to and rates of acquisition/reacquisition, and carriage duration and clearance rates of different strains. For some pathogens, such as *S. pneumoniae*, the distribution of strain types or serotypes varies geographically (10). Therefore, studies to describe carriage dynamics should be conducted in different countries to account for the geographical heterogeneity, which may result in accurate inferences from the models.

Assessment of pneumococcal carriage dynamics requires conducting both cross-sectional and longitudinal studies. Cross-sectional carriage surveys are generally easier and cheaper to conduct; therefore, are widely used to provide a snapshot of pneumococcal populations. Conversely, longitudinal studies are usually prohibitively expensive to conduct since they require following up the participants for a period of time. Few high-quality longitudinal studies on pneumococcal carriage have been conducted to date in infants from The Gambia (11, 12), Kenya (13), South Africa (14), Thailand (15), and Papua New Guinea (16). However, due to the geographical differences in serotype distribution between settings, additional studies are required to capture the characteristics of ≥100 known pneumococcal serotypes and to better understand their geographical stability, and heterogeneity between different settings (17, 18). Furthermore, in some settings such as The Gambia, the majority of the pneumococcal carriage studies have focused on describing the carriage dynamics of only a few of the most common serotypes (11, 12).

In this study, we used serotyping data from longitudinally sampled pneumococcal isolates to assess acquisition, reacquisition, carriage duration and clearance of serotypes during the first year of life in newborn infants from a rural setting in the Gambia, West Africa. Previous studies have shown high carriage rates reaching 100% among infants in the Gambia, which makes this setting ideal for this study (19).

## Methods

### Study Design and Participants

The Sibanor Nasopharyngeal Microbiome (SNM) study collected nasopharyngeal swabs (*n* = 1,595) from 102 newborn infants from 21 villages in the Gambia between September 2008 and April 2009 as previously described (20). The infants were from communities with and without exposure to the seven-valent PCV (PCV7). Parents and guardians of the infants gave informed consent before enrolling them into the study. Nasopharyngeal swabs were then taken from the infants starting from the first week after birth followed by swabs every 2 weeks until 6 months (weeks 1, 3, 5 to 27) and then every two months until their first birthday (weeks 35, 43, and 52). Two-thirds of the infants received PCV7 at 2, 3, and 4 months, and the remaining third received at least 1 dose of PCV during the PCV7 implementation nationwide catch-up campaign. Nasopharyngeal (NPS) specimens were stored in skim milk–tryptone-glucose glycerol medium (STGG) and stored at −80°C within 8 hours of collection.

### Sample Processing, Serotyping, and Sequencing

For the isolation of *S. pneumoniae*, broth enrichment of nasopharyngeal swab samples (NPS) using 5 mL of Todd-Hewitt broth (Oxoid, Basingstoke, UK) containing 5% yeast extract with 1 mL rabbit serum (TCS Biosciences Ltd, Botolph Claydon, UK) was performed as described elsewhere (19). We identified pneumococci by their morphology and optochin sensitivity. Sterile saline suspensions of pneumococcal plate sweeps (on gentamicin blood agar) were then used for serotyping by latex agglutination which can detect multiple serotypes (15). Latex agglutination was performed by capsular and factor-typing sera (Statens Serum Institut, Copenhagen, Denmark) as described previously (21). We also utilized genotypically determined serotype information inferred from whole genome sequencing data for some of the NPS samples as previously described (22) through a collaboration with the Global Pneumococcal Sequencing (GPS) project (www.pneumogen.net) (23).

### Statistical Analysis

We defined a colonization episode to be either the first acquisition or re-acquisition of a serotype after clearance; whereby clearance is referred to as the detection of negative cultures for any serotype at two consecutive sampling points as previously described (15). For episodes which commenced at the first week after birth and terminating before week 27, acquisition and clearance of the serotype was assumed to occur at the mid-point between consecutive sampling points. From week 35 to week 52 where samples were collected bi-monthly, acquisition and clearance of serotypes was assumed to occur 1 week before and after detection to prevent overestimation of the carriage duration.

We then fitted a time-to-event parametric model with constant hazard rate (exponential distribution) to the data on time until clearance of serotypes, to estimate the clearance rate as the hazard rate of each serotype. The carriage duration was calculated as the inverse of the hazard rate. Similarly, the acquisition rate and time to acquisition for the first and second carriage episodes with a serotype were estimated as the hazard rate and its inverse after fitting the parametric model to the time until first acquisition data for each serotype. The differences between time-to-event estimates were assessed using log-rank test using non-parametric time-to-event models. We used the survival version 2.42.3 to fit the parametric time-to-event model (24, 25).

We also obtained point estimates for the carriage duration of serotypes in other countries where similar studies were conducted, namely Kenya (13) and South Africa (14). Correlation between the estimates were assessed using the Pearson's correlation coefficient (ρ). The median differences between carriage durations were assessed using Wilcoxon test for paired samples. Graphs were plotted using ggplot2 version 3.3.2 (26) while network visualization was done using Cytoscape version 3.8.1 (27). All the statistical analyses were conducted in R version 3.5.3 (28).

## Results

**Figure 1:**
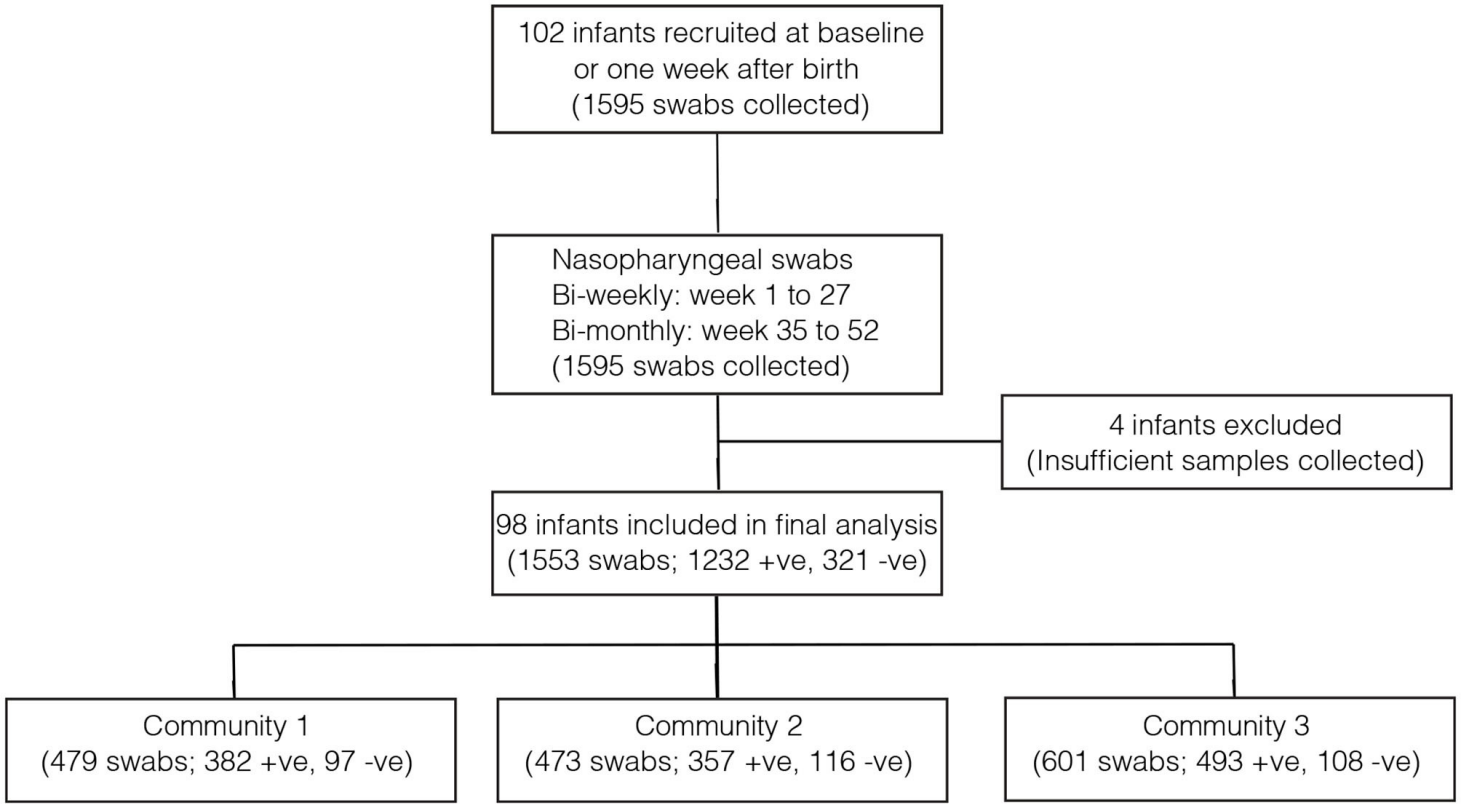
Flow diagram showing the number of infants and pneumococcal samples included in the analysis. The infants in community 1 were from PCV-unexposed (unvaccinated) villages and received PCV7 after 6 months (control group). The infants in community 2 were also from PCV-unexposed (unvaccinated) and they received PCV7 at 2, 3, and 4 months (direct impact of vaccination). The infants from community 3 came from PCV-exposed (vaccinated) villages and they received PCV7 at 2, 3, and 4 months (direct impact of vaccination and herd immunity).

**Figure 2:**
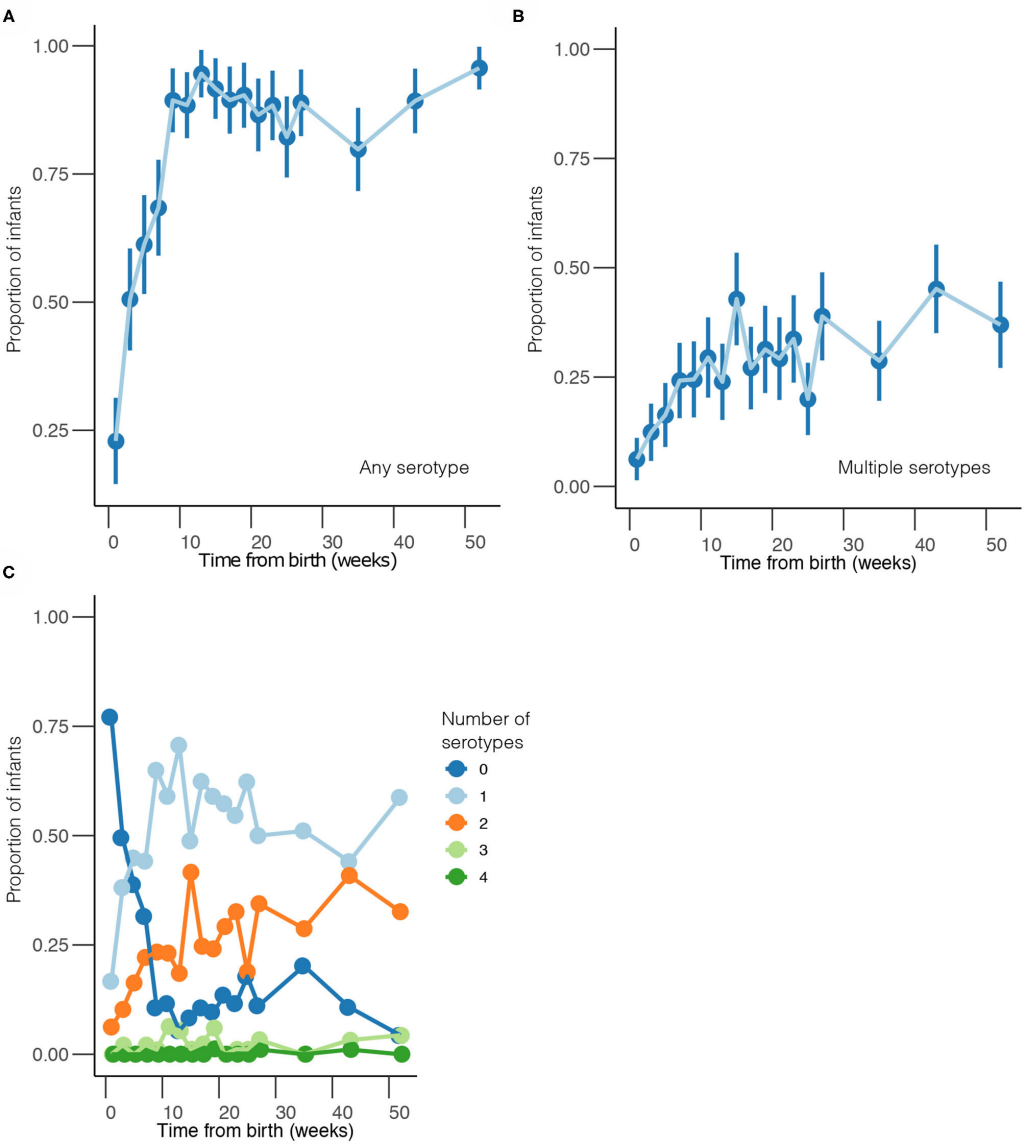
Temporal prevalence of pneumococcal serotypes during the first year of life. **(A)** Proportion of infants colonized with any serotype at each sampling point. **(B)** Proportion of infants co-colonized with multiple serotypes at each sampling point. **(C)** Proportion of infants colonized by a different number of serotypes at each sampling point. The estimates are shown at bi-weekly sampling points from weeks 1 to 27, and then bi-monthly sampling intervals from weeks 35 to 52.

**Figure 3:**
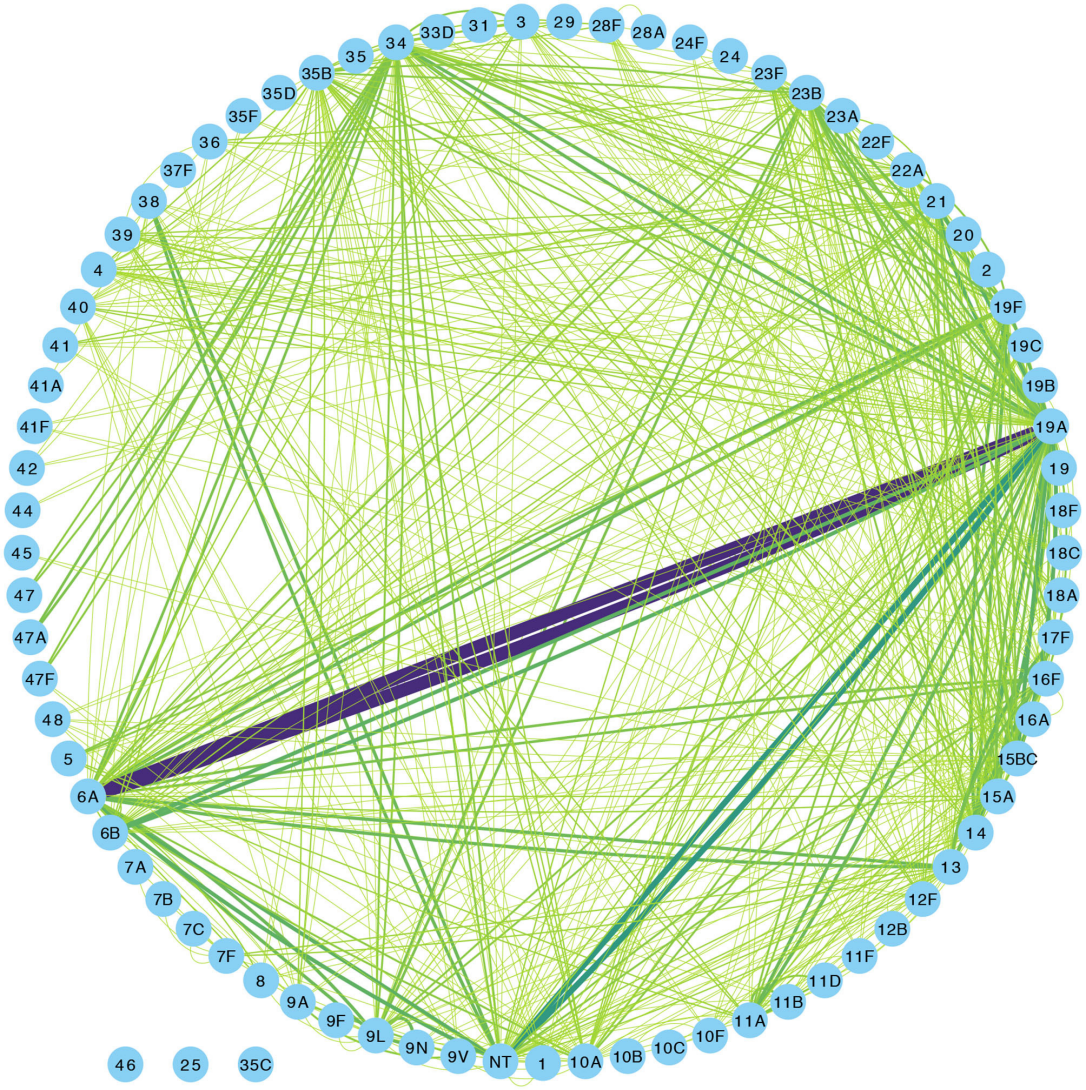
Network graph showing co-colonization of pneumococcal strains among Gambian infants. The width of the edges connecting a pair of nodes (or serotypes) in the network is proportional to the number of co-occurrences of the pair of serotypes at the same sampling point in the same infant. The shades of color from light green to indigo for each connecting line in the network correspond to the number of detected serotype co-occurrences ranging from 0 to 20.

Overall, 1,553 samples from 98 out of 102 newborn infants recruited in the SNM study were analyzed (Figure 1 and Table 1). We identified 80 pneumococcal serotypes naturally colonizing the infants, and pneumococcal-culture positivity rate was 79.3% (1,232/1,553). The mean number of serotypes detected per infant was ≈9 (range: 3–15) while an average of ≈9 carriage episodes (range: 2–15) were detected per infant. By counting each serotype once per episode, serotypes associated with the most carriage episodes were 19A (11.4%), 6A (8.74%), non-typeable strains (NT) (5.71%), 15B/C (4.90%), 19F (3.85%), 23B (4.31%), 34 (3.61%), 21 (3.26%), 35B (3.26%), and 11A (3.03%) (Supplementary Figure 1). All the infants experienced at least 1 carriage episode during the first year of life. Approximately 31% of the infants were colonized within the first week of life but the prevalence increased rapidly to 95% by 2 months and nearly 100% by 12 months (Figure 2A). We then assessed the temporal prevalence of colonization with multiple serotypes during the first year of life. The multiple colonization rate was >40% by 12 months (Figures 2B,C). The proportion of infants carrying multiple serotypes started to plateau approximately 10 weeks after birth mirroring the pattern seen for the overall prevalence of serotypes colonizing the infants. Some serotypes appeared to co-colonize the same infant, for example 6A and 19A, NT and 38, 23B and 9L, 6B and NT (Figure 3).

**Table 1:** Characteristics of the infants enrolled in the study.

**Characteristic**		**Frequency (%)**
Sex	Male	52.04 (51/98)
	Female	47.96 (47/98)
Mother's age	16–20	18.37 (18/98)
	21–30	45.92 (45/98)
	31–40	35.71 (35/98)
Place of birth	Health center	14.29 (14/98)
	Home	51.02 (50/98)
	Hospital	34.69 (34/98)
Type of birth	Vaginal	100.00 (98/98)
Timing of birth	Full term	98.98 (97/98)
	Premature	1.02 (1/98)
Vaccination^*^	Community 1	32.65 (32/98)
	Community 2	29.59 (29/98)
	Community 3	37.76 (37/98)
Number of siblings	0	14.29 (14/98)
	1	16.33 (16/98)
	2	19.39 (19/98)
	3	12.24 (12/98)
	4–9	37.76 (37/98)
Vaccinated sibling	Yes	5.10 (5/98)
	No	94.90 (93/98)

* *The infants in community 1 were from PCV-unexposed (unvaccinated) villages and received PCV7 after 6 months (control group). The infants in community 2 were also from PCV-unexposed (unvaccinated) and they received PCV7 at 2, 3, and 4 months (direct impact of vaccination). The infants from community 3 came from PCV-exposed (vaccinated) villages and they received PCV7 at 2, 3, and 4 months (direct impact of vaccination and herd immunity)*.

**Figure 4:**
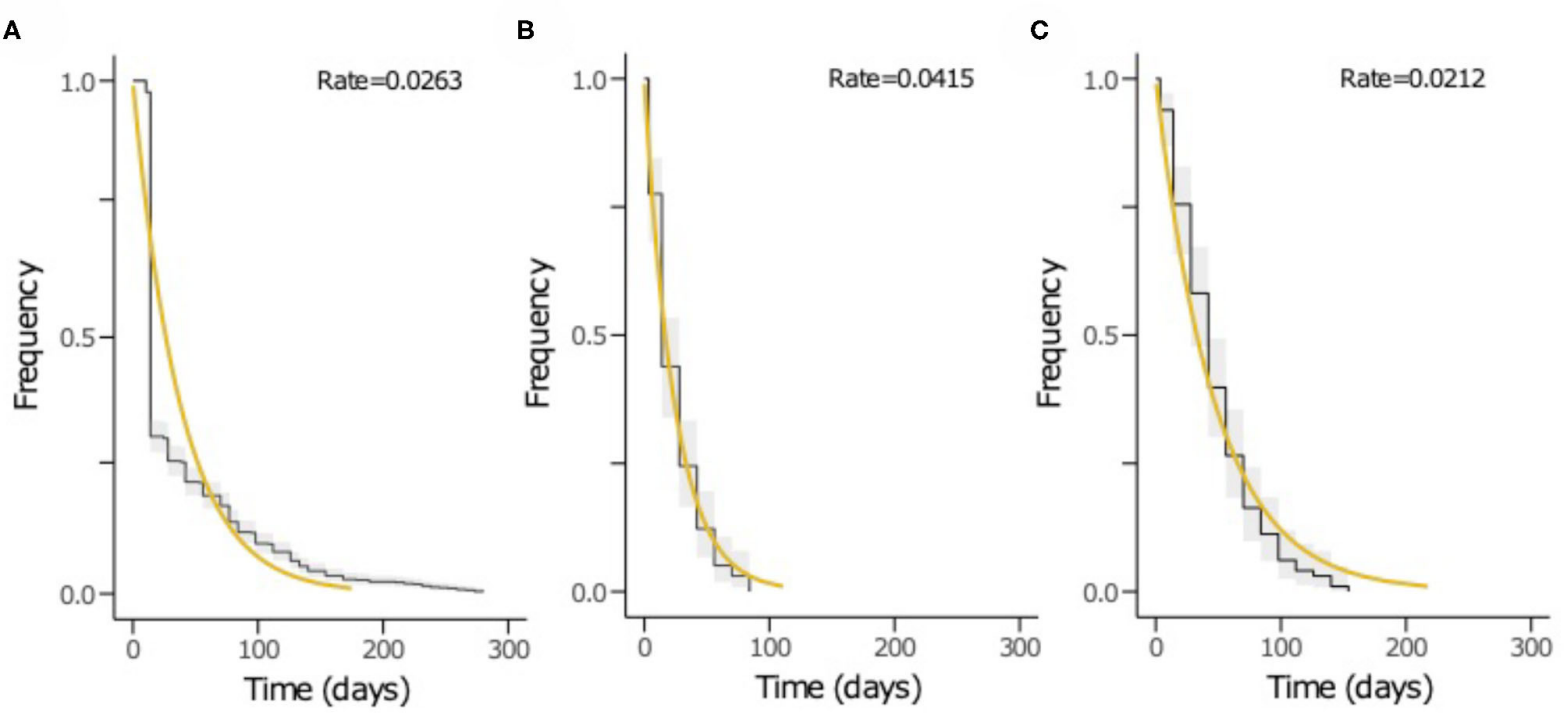
The Kaplan-Meier survival curves and exponential fit to the longitudinal pneumococcal carriage data. The plots show **(A)** duration from acquisition to clearance of all serotypes, **(B)** time until first acquisition of any serotype and **(C)** time until second acquisition or reacquisition of any serotype. The black curve represents the Kaplan-Meier estimates while the yellow line is the fitted survival curve. The rate parameter in the exponential model is shown at the top of the plots **(A–C)** represents the mean clearance, acquisition and reacquisition rate, respectively. The inverse of the rate in plots A, B and C equates to mean the carriage duration, time until first acquisition and reacquisition of any serotype, respectively.

The time to acquisition of any serotype was 24.1 days [95% confidence interval (CI): 19.7–29.3], and the overall carriage duration was 38.0 days (95% CI: 35.5–40.6) (Figures 4A–C). These implied overall pneumococcal acquisition and clearance rates of 0.042 (95% CI: 0.034–0.051) and 0.026 (95% CI: 0.025–0.028) episodes/day, respectively. We then assessed clearance, acquisition and the reacquisition of individual serotypes (Figures 4A–C, Supplementary Table 1 and Supplementary Figures 2–8). The mean carriage duration of different serotypes ranged from 13.9 to 69.3 days. Serotypes 6B, 4, 13, 23F, and 19F showed longest the carriage durations, and therefore, lowest clearance rates while serotypes with the shortest carriage durations included 1, 5, and 47F (Table 2). The quickest time to first acquisition was associated with serotypes 18, 48, and 47 in contrast delayed acquisition was associated with serotypes 10A, 19C, and 16A.

**Table 2:** Carriage duration, initial acquisition and reacquisition times of pneumococcal serotypes in the among Gambian infants.

**Serotype**	**Number of episodes**	**Prevalence (%)**	**Carriage duration in days**			**Time from birth to first acquisition in days (95% CI)**	**Time from birth to reacquisition in days (95% CI)**
			**All episodes (95% CI)**	**First episode (95% CI)**	**Second episode (95% CI)**		
47F	3	0.35	12.83 (4.14, 39.79)	12.83 (4.14, 39.79)	–	143.5 (46.28, 444.93)	–
41	3	0.35	14 (4.52, 43.41)	–	–	–	–
19B	4	0.47	14 (5.25, 37.3)	14 (5.25, 37.3)	–	159.25 (59.77, 424.31)	–
47	4	0.47	14 (5.25, 37.3)	14 (4.52, 43.41)	–	98 (31.61, 303.86)	–
47A	4	0.47	14 (5.25, 37.3)	14 (5.25, 37.3)	–	73.5 (27.59, 195.83)	–
5	4	0.47	14 (5.25, 37.3)	14 (5.25, 37.3)	–	189 (70.94, 503.57)	–
10F	5	0.58	14 (5.83, 33.64)	14 (5.83, 33.64)	–	144.2 (60.02, 346.44)	–
16A	5	0.58	14 (5.83, 33.64)	14 (5.83, 33.64)	–	266 (110.72, 639.07)	–
36	6	0.7	14 (6.29, 31.16)	14 (6.29, 31.16)	–	79.33 (35.64, 176.59)	–
19C	11	1.28	14 (7.75, 25.28)	14 (7.75, 25.28)	–	211.27 (117, 381.5)	–
1	8	0.93	15.75 (7.88, 31.49)	15.75 (7.88, 31.49)	–	94.5 (47.26, 188.96)	–
9N	7	0.82	16 (7.63, 33.56)	16.33 (7.34, 36.36)	–	175 (78.62, 389.53)	–
8	5	0.58	16.8 (6.99, 40.36)	16.8 (6.99, 40.36)	–	112 (46.62, 269.08)	–
28F	7	0.82	17.5 (8.34, 36.71)	17.5 (8.34, 36.71)	–	110.5 (52.68, 231.79)	–
NT	49	5.71	19.79 (14.95, 26.18)	21.46 (15.62, 29.49)	14 (7.75, 25.28)	107.67 (78.35, 147.97)	233.55 (129.34, 421.71)
35F	4	0.47	21 (7.88, 55.95)	21 (7.88, 55.95)	–	168 (63.05, 447.62)	–
11D	3	0.35	23.33 (7.53, 72.35)	23.33 (7.53, 72.35)	–	182 (58.7, 564.3)	–
18C	7	0.82	24 (11.44, 50.34)	25.67 (11.53, 57.13)	–	79.33 (35.64, 176.59)	–
3	8	0.93	24.94 (12.47, 49.87)	17.5 (8.34, 36.71)	–	116.5 (55.54, 244.37)	–
11B	8	0.93	26.25 (13.13, 52.49)	28 (13.35, 58.73)	–	190 (90.58, 398.55)	–
38	9	1.05	26.44 (13.76, 50.82)	24.5 (12.25, 48.99)	–	145.25 (72.64, 290.44)	–
23A	13	1.52	26.92 (15.63, 46.37)	28 (15.9, 49.3)	–	116.08 (65.92, 204.4)	–
12F	8	0.93	28 (14, 55.99)	28 (14, 55.99)	–	117.25 (58.64, 234.45)	–
40	11	1.28	29.27 (16.21, 52.86)	30.8 (16.57, 57.24)	–	154.7 (83.24, 287.52)	–
9V	8	0.93	29.75 (14.88, 59.49)	32 (15.26, 67.12)	–	169 (80.57, 354.5)	–
7F	4	0.47	31.5 (11.82, 83.93)	31.5 (11.82, 83.93)	–	187.25 (70.28, 498.91)	–
17F	11	1.28	31.5 (17.44, 56.88)	31.85 (17.14, 59.19)	–	196.35 (105.65, 364.93)	–
18A	6	0.7	32.08 (14.41, 71.41)	32.08 (14.41, 71.41)	–	51.92 (23.32, 115.56)	–
48	6	0.7	32.67 (14.68, 72.71)	36.4 (15.15, 87.45)	–	70 (29.14, 168.18)	–
34	31	3.61	34.1 (23.98, 48.48)	35.48 (24.66, 51.06)	–	117.31 (81.52, 168.81)	–
9A	7	0.82	35 (16.69, 73.42)	35 (16.69, 73.42)	–	118 (56.25, 247.52)	–
19F	33	3.85	35.64 (25.33, 50.13)	35 (24.47, 50.06)	42 (13.55, 130.22)	168.47 (117.79, 240.95)	231 (74.5, 716.23)
15A	18	2.1	35.78 (22.54, 56.79)	29.65 (18.43, 47.69)	–	146.18 (90.87, 235.14)	
15B/C	42	4.9	36.17 (26.73, 48.94)	32.28 (23.28, 44.75)	59.5 (26.73, 132.44)	181.42 (130.86, 251.5)	249.67 (112.17, 555.73)
16F	24	2.8	36.9 (24.73, 55.05)	29.75 (19.59, 45.18)	–	153.84 (101.3, 233.64)	–
22A	12	1.4	38.5 (21.86, 67.79)	35 (18.83, 65.05)	–	114.8 (61.77, 213.36)	–
14	25	2.91	38.5 (26.01, 56.98)	35.18 (22.44, 55.16)	49 (22.01, 109.07)	119.92 (76.49, 188.01)	123.2 (51.28, 295.99)
23B	32	3.73	40.47 (28.62, 57.23)	34.74 (23.82, 50.66)	71.4 (29.72, 171.54)	113.56 (77.87, 165.59)	173.6 (72.26, 417.08)
10A	17	1.98	41.18 (25.6, 66.24)	44.8 (27.01, 74.31)	–	196.93 (118.72, 326.66)	–
11A	26	3.03	41.46 (28.23, 60.89)	24.82 (16.34, 37.69)	133 (49.92, 354.37)	168.95 (111.25, 256.59)	206.5 (77.5, 550.2)
6A	75	8.74	43.12 (34.39, 54.07)	45.03 (34.97, 58)	35.47 (21.38, 58.83)	110.72 (85.97, 142.59)	199.5 (118.15, 336.85)
21	28	3.26	43.38 (29.95, 62.82)	41.85 (28.05, 62.44)	52.5 (19.7, 139.88)	117.98 (79.08, 176.02)	–
35B	28	3.26	46.5 (32.11, 67.35)	38.92 (26.3, 57.6)	109.67 (35.37, 340.03)	166.88 (112.76, 246.97)	126 (40.64, 390.67)
39	7	0.82	47 (22.41, 98.59)	38.5 (17.3, 85.7)	–	130.67 (58.7, 290.85)	–
20	10	1.17	47.95 (25.8, 89.12)	56.44 (28.22, 112.85)	–	115.94 (57.98, 231.83)	–
9L	15	1.75	54.13 (32.64, 89.79)	54.25 (30.81, 95.53)	53.67 (17.31, 166.4)	99.17 (56.32, 174.62)	–
19A	98	11.42	56.18 (46.09, 68.48)	63.53 (50.43, 80.04)	35.81 (24.38, 52.59)	134.02 (106.38, 168.84)	181 (118.01, 277.6)
23F	14	1.63	57.5 (34.05, 97.09)	59.18 (32.77, 106.86)	51.33 (16.56, 159.16)	105.64 (58.5, 190.75)	221.67 (71.49, 687.29)
13	24	2.8	59.5 (39.88, 88.77)	57.27 (37.71, 86.98)	–	160.36 (105.59, 243.55)	–
4	5	0.58	63 (26.22, 151.36)	63 (26.22, 151.36)	–	176.4 (73.42, 423.81)	–
6B	17	1.98	85.85 (53.37, 138.1)	90.25 (53.45, 152.38)	65.33 (21.07, 202.57)	84.25 (49.9, 142.25)	149.33 (48.16, 463.02)

*Cells marked by “–” designate serotypes where no or very few data points (<3) were available for analysis*.

**Figure 5:**
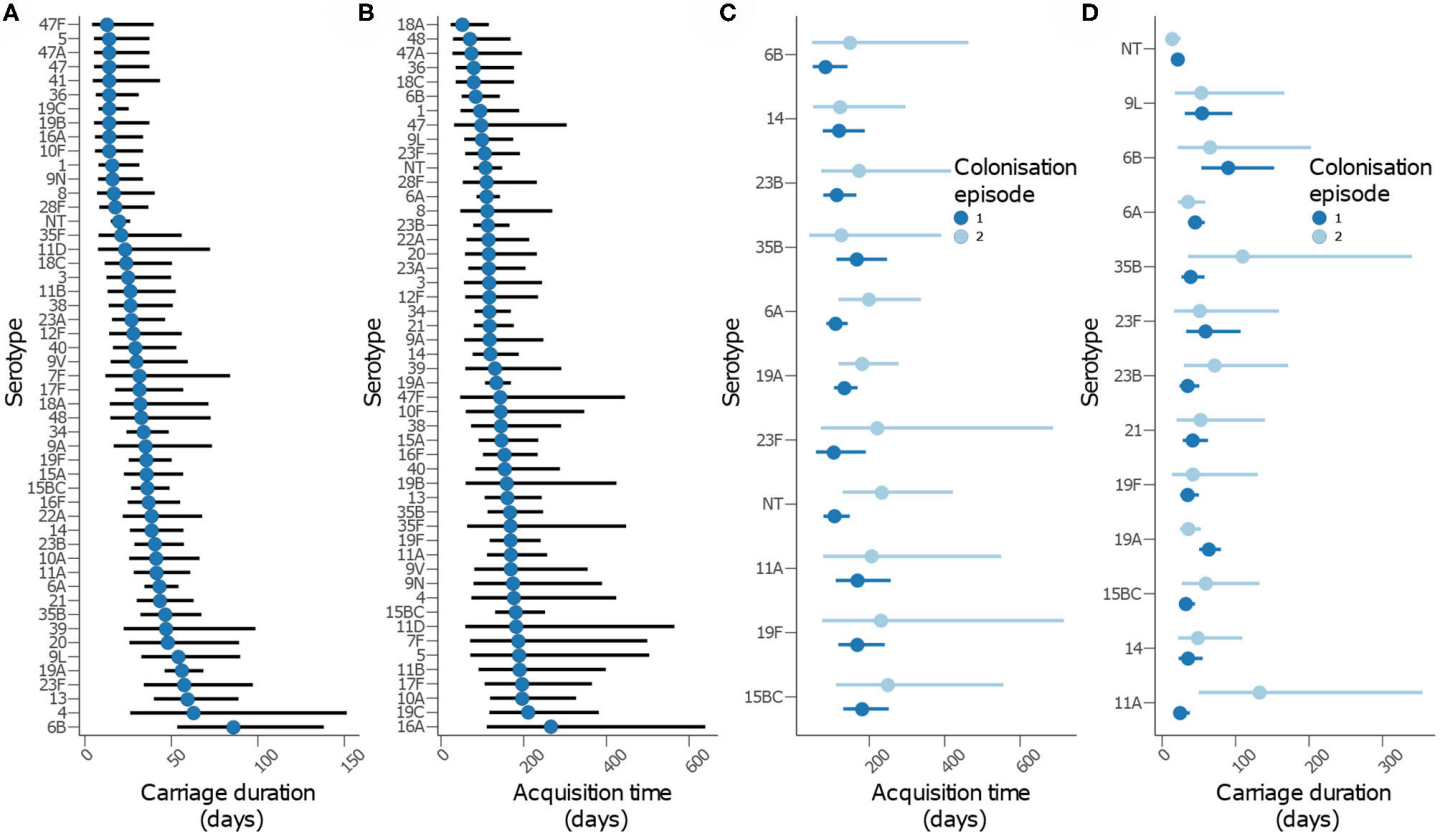
Carriage dynamics of pneumococcal serotypes in Gambian infants. Graph showing **(A)** carriage duration, **(B)** time to first acquisition, **(C)** time to acquisition from birth for the first and second colonization episode with the serotype, **(D)** carriage duration for the first and second colonization episode with the serotype.

We then assessed whether time to acquisition and reacquisition of serotypes from birth were similar (Figure 5 and Table 2, Supplementary Table 1 and Supplementary Figure 9). There were no differences in the time to acquisition and reacquisitions of the same serotype from birth were similar except for serotypes 6A, 23F, and NT, which are known pediatric serotypes (29, 30) (Figure 5C and Supplementary Figure 10C). Similarity of the carriage duration after initial colonization was also investigated. No differences were observed for all serotypes except for serotype 11A, which showed longer carriage durations after reacquisition as compared to the initial (*P* = 0.0026) (Figure 5D and Supplementary Figure 10D). Furthermore, the carriage duration and time to first acquisition of serotypes between PCV-exposed and unexposed communities were also similar (Supplementary Figures 9, 10).

**Figure 6:**
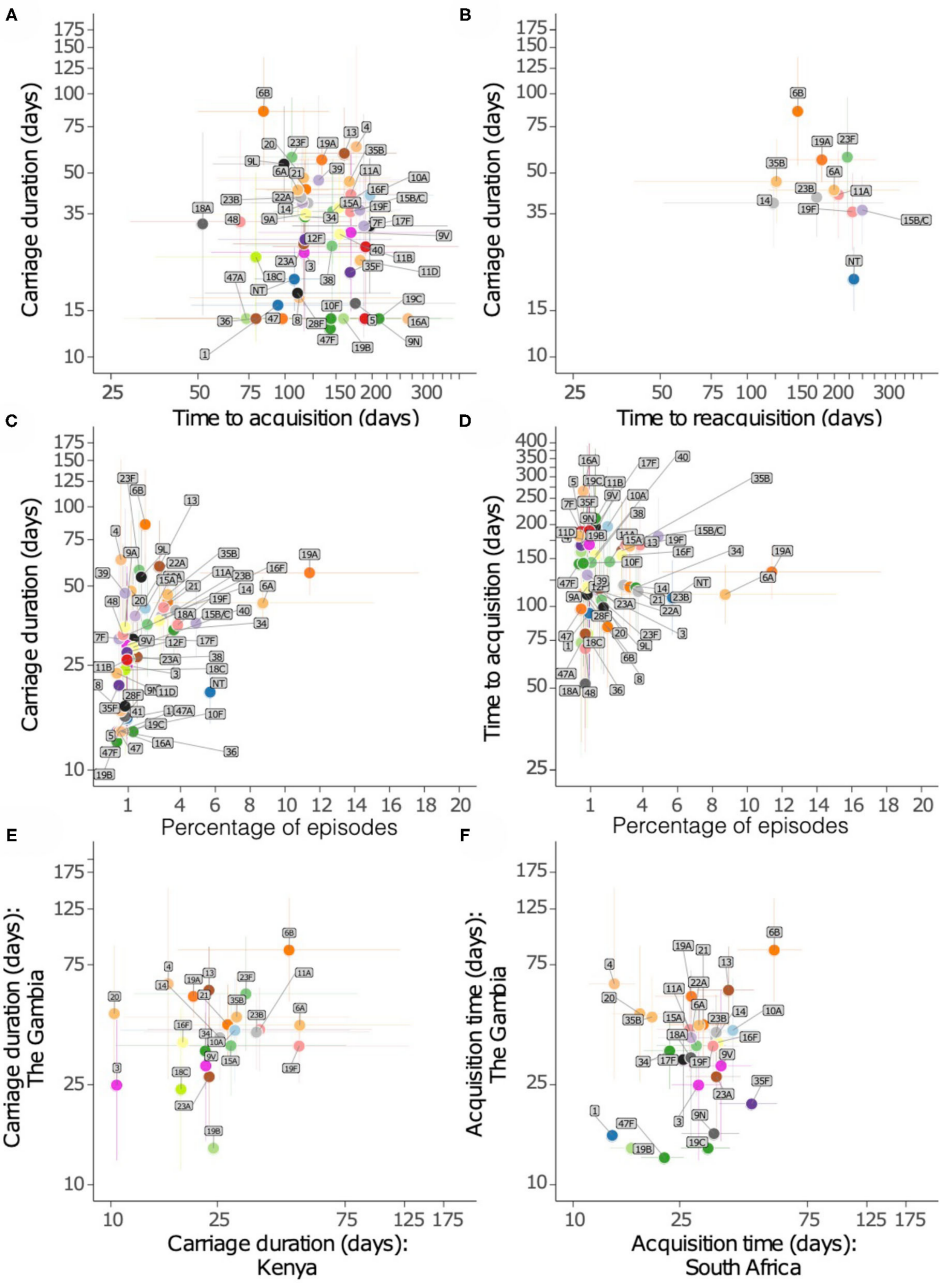
Relationship of carriage estimates of pneumococcal serotypes in Gambian infants. **(A)** Scatter plot showing carriage duration and time to first acquisition. **(B)** Scatter plot showing carriage duration, second acquisition (reacquisition). **(C)** Scatter plot showing carriage duration and frequency of serotype/episode, **(D)** time to first acquisition and frequency of serotypes. **(E)** Scatter plot showing carriage duration of serotypes in the Gambia and Kenya. **(F)** Scatter plot showing time to acquisition of serotypes in the Gambia and South Africa.

We then assessed whether serotypes acquired earliest had the longest carriage duration (Figures 6A,B). There was no association between carriage duration and time to first acquisition (ρ = −0.127, *P* = 0.381). In contrast, serotypes which were more prevalent were likely to be carried for longer durations than those carried for short durations (ρ = 0.404, *P* = 0.003), but there was no association between carriage prevalence and time to initial acquisition of serotypes (ρ = −0.047, *P* = 0.745) (Figures 6C,D).

We compared the points estimates for the carriage duration of serotypes in the present study and similar studies in infants from Kenya (13) and South Africa (14) (Figures 6E,F). The carriage duration estimates from Thailand were available for few serotypes only as such were excluded (15). A weak correlation was observed between carriage duration of serotypes between the Gambia and South Africa (ρ = 0.302, *P* = 0.118), and the Gambia and Kenya (ρ = 0.357, *P* = 0.094).

## Discussion

We have described the carriage duration and acquisition patterns of pneumococcal serotypes during the first year of life in the Gambia. We show high and rapid colonization of newborn infants, one third of which carried multiple serotypes. Colonization rates exceeded 20% one week after birth mostly due to serotypes 6A, 34, and NTs. Such early acquisition of certain serotypes may reflect rapid loss of maternally derived immunity (31, 32). Carriage duration of serotypes varied greatly but serotypes carried for longer duration such as 6B were not necessarily acquired earlier in life than serotypes carriage for short durations such as serotype 1. We also found that time from birth to first acquisition and to first reacquisition from birth were similar, which suggests continuous exposure leading to reacquisition of serotypes, possibly among household contacts. The absence of differences for the time to acquisition and carriage duration of serotypes among the infants from vaccinated, vaccine-exposed and unvaccinated communities could be indicative of relatively low herd immunity from PCV7 roll-out at the time of the study. Our findings provide a detailed description of the pneumococcal carriage dynamics in The Gambia, which complements previous pneumococcal carriage studies in the same setting (11, 12).

Since the prevalence of serotypes in carriage varies between countries (10), this may affect how the strains interact and compete in different settings, which may influence carriage dynamics within hosts. By comparing durations between the Gambia (this study), Kenya (13) and South Africa (14), we show that carriage duration is stable geographically for the majority of serotypes although substantial heterogeneity was observed for some serotypes, potentially reflecting variability in local selective pressures. Our study also provides carriage duration estimates for serotypes whose carriage duration were previously unknown in our setting including serotypes 5, 7F, 39, 9A, 40, 28F, and 12F (13–15). Availability of this information will lead to reliable inferences for mathematical models for assessing the population effects of clinical interventions such as PCVs and antibiotics in our geographical setting.

Our study has some limitations. Although our serotyping approach detected colonization with multiple serotypes, it did not quantify their abundance and may have missed less abundant serotypes. To generate a better picture of co-colonization of serotypes, future studies should utilize other high resolution techniques including microarray and deep sequencing of plate sweeps enriched for the pneumococcus (33, 34). Due to the small sample size, our study was not powered to detect potential synergistic or antagonistic interactions between co-carried serotypes on carriage duration of each serotype. In addition, larger sample sizes are needed to obtain robust estimates for rare serotypes. Lastly, the observed carriage duration of serotypes sampled at non-consecutive time points such as serotypes 1, 5, 16A, 41, 19C, 9N, and 47F may have been slightly overestimated. This may be due to the assumption that colonization episodes start and terminate at the midpoint between consecutive sampling periods i.e., acquisition at 1 week before detection and 1 week after last detection. Therefore, caution is required when interpreting the estimated carriage duration for these serotypes.

The implementation of higher-valency PCVs has led to remarkable changes in the pneumococcal populations in Sub Saharan Africa (35–38) and globally (39–42). Our study provides the first comprehensive analysis of temporal carriage dynamics of pneumococcal serotypes in West Africa, which will enhance our understanding of the epidemiology in infants, an age group associated with the greatest risk of pneumococcal diseases and mortality, to inform future prevention and control strategies. Furthermore, our findings will provide baseline data for parameterising mathematical models for infectious diseases to assess and forecast population-level effects of PCVs and antibiotic resistance in the region.

## Data Availability Statement

The datasets generated for this study can be found in online repositories. The names of the repository/repositories and accession number(s) can be found in the article/Supplementary Material.

## Ethics Statement

The SNM study was approved by the Medical Research Council Unit The Gambia and the Gambian Government Joint Ethics Committee (approval number: SCC1108). The patients/participants provided their written informed consent to participate in this study.

## Author Contributions

BK-A, MA, and RA conducted field and sample collection activities in the Sibanor Nasopharyngeal Microbiome (SNM) study. CC, MS, and BK-A conceived and planned the analysis. The Global Pneumococcal Sequencing (GPS) project was led by KK, RBr, LM, and SB. RG and SL performed genome-based serotyping and quality control of sequenced isolates. P-ET, EF-N, RBa, FC, and CO performed microbiology work. CC carried out the analysis. CC and BK-A drafted the initial version of the manuscript. CC, MS, EB, SL, CE, RG, P-ET, RBa, AW, EF-N, FC, CO, LM, KK, RBr, MB, RA, MA, SB, and BK-A reviewed and contributed to discussions of the manuscript. All authors contributed to the article and approved the submitted version.

## Conflict of Interest

The authors declare that the research was conducted in the absence of any commercial or financial relationships that could be construed as a potential conflict of interest.
