# Supplementary material for: Carriage Dynamics of Pneumococcal Serotypes in Naturally Colonized Infants in a Rural African Setting During the First Year of Life
Source: Front Pediatr. 2021 Jan 8;8:587730. doi: 10.3389/fped.2020.587730 (PMC7820366; doi:10.3389/fped.2020.587730)
Supplement: Supplementary file 1 [file Data_Sheet_1.PDF]

Carriage dynamics of pneumococcal serotypes in naturally colonised infants in a rural African setting during the first year of life

Chrispin Chaguza, Madikay Senghore, Ebrima Bojang, Stephanie W. Lo, Chinelo Ebruke, Rebecca A. Gladstone, Peggy-Estelle Tientcheu, Rowan E. Bancroft, Archibald Worwui, Ebenezer Foster-Nyarko, Fatima Ceesay, Catherine Okoi, Lesley McGee, Keith P. Klugman, Robert F. Breiman, Michael R. Barer, Richard A. Adegbola, Martin Antonio, Stephen D. Bentley, and Brenda A. Kwambana-Adams

Supplementary Table 1. Clearance and acquisition rates of pneumococcal serotypes in the Gambian infants.

Serotype	Clearance rate (per day)			Time from birth to first acquisition in days (95% CI)	Time from birth to reacquisition in days (95% CI)
	All episodes (95% CI)	First episode (95% CI)	Second episode (95% CI)		
**6B**	0.0116 (0.0072,0.0187)	0.0111 (0.0066,0.0187)	0.0153 (0.0049,0.0475)	0.0119 (0.007,0.02)	0.0067 (0.0022,0.0208)
**4**	0.0159 (0.0066,0.0381)	0.0159 (0.0066,0.0381)	–	0.0057 (0.0024,0.0136)	–
**13**	0.0168 (0.0113,0.0251)	0.0175 (0.0115,0.0265)	–	0.0062 (0.0041,0.0095)	–
**23F**	0.0174 (0.0103,0.0294)	0.0169 (0.0094,0.0305)	0.0195 (0.0063,0.0604)	0.0095 (0.0052,0.0171)	0.0045 (0.0015,0.014)
**19A**	0.0178 (0.0146,0.0217)	0.0157 (0.0125,0.0198)	0.0279 (0.019,0.041)	0.0075 (0.0059,0.0094)	0.0055 (0.0036,0.0085)
**9L**	0.0185 (0.0111,0.0306)	0.0184 (0.0105,0.0325)	0.0186 (0.006,0.0578)	0.0101 (0.0057,0.0178)	–
**20**	0.0209 (0.0112,0.0388)	0.0177 (0.0089,0.0354)	–	0.0086 (0.0043,0.0172)	–
**39**	0.0213 (0.0101,0.0446)	0.026 (0.0117,0.0578)	–	0.0077 (0.0034,0.017)	–
**35B**	0.0215 (0.0148,0.0311)	0.0257 (0.0174,0.038)	0.0091 (0.0029,0.0283)	0.006 (0.004,0.0089)	0.0079 (0.0026,0.0246)
**21**	0.0231 (0.0159,0.0334)	0.0239 (0.016,0.0356)	0.019 (0.0071,0.0508)	0.0085 (0.0057,0.0126)	–
**6A**	0.0232 (0.0185,0.0291)	0.0222 (0.0172,0.0286)	0.0282 (0.017,0.0468)	0.009 (0.007,0.0116)	0.005 (0.003,0.0085)
**11A**	0.0241 (0.0164,0.0354)	0.0403 (0.0265,0.0612)	0.0075 (0.0028,0.02)	0.0059 (0.0039,0.009)	0.0048 (0.0018,0.0129)
**10A**	0.0243 (0.0151,0.0391)	0.0223 (0.0135,0.037)	–	0.0051 (0.0031,0.0084)	–
**23B**	0.0247 (0.0175,0.0349)	0.0288 (0.0197,0.042)	0.014 (0.0058,0.0336)	0.0088 (0.006,0.0128)	0.0058 (0.0024,0.0138)
**22A**	0.026 (0.0148,0.0457)	0.0286 (0.0154,0.0531)	–	0.0087 (0.0047,0.0162)	–
**14**	0.026 (0.0176,0.0384)	0.0284 (0.0181,0.0446)	0.0204 (0.0092,0.0454)	0.0083 (0.0053,0.0131)	0.0081 (0.0034,0.0195)
**16F**	0.0271 (0.0182,0.0404)	0.0336 (0.0221,0.051)	–	0.0065 (0.0043,0.0099)	–
**15B/C**	0.0276 (0.0204,0.0374)	0.031 (0.0223,0.043)	0.0168 (0.0076,0.0374)	0.0055 (0.004,0.0076)	0.004 (0.0018,0.0089)
**15A**	0.028 (0.0176,0.0444)	0.0337 (0.021,0.0543)	–	0.0068 (0.0043,0.011)	–
**19F**	0.0281 (0.0199,0.0395)	0.0286 (0.02,0.0409)	0.0238 (0.0077,0.0738)	0.0059 (0.0042,0.0085)	0.0043 (0.0014,0.0134)
**9A**	0.0286 (0.0136,0.0599)	0.0286 (0.0136,0.0599)	–	0.0085 (0.004,0.0178)	–
**34**	0.0293 (0.0206,0.0417)	0.0282 (0.0196,0.0406)	–	0.0085 (0.0059,0.0123)	–
**48**	0.0306 (0.0138,0.0681)	0.0275 (0.0114,0.066)	–	0.0143 (0.0059,0.0343)	–
**18A**	0.0312 (0.014,0.0694)	0.0312 (0.014,0.0694)	–	0.0193 (0.0087,0.0429)	–
**7F**	0.0317 (0.0119,0.0846)	0.0317 (0.0119,0.0846)	–	0.0053 (0.002,0.0142)	–
**17F**	0.0317 (0.0176,0.0573)	0.0314 (0.0169,0.0584)	–	0.0051 (0.0027,0.0095)	–
**9V**	0.0336 (0.0168,0.0672)	0.0313 (0.0149,0.0656)	–	0.0059 (0.0028,0.0124)	–
**40**	0.0342 (0.0189,0.0617)	0.0325 (0.0175,0.0603)	–	0.0065 (0.0035,0.012)	–
**12F**	0.0357 (0.0179,0.0714)	0.0357 (0.0179,0.0714)	–	0.0085 (0.0043,0.0171)	–
**23A**	0.0371 (0.0216,0.064)	0.0357 (0.0203,0.0629)	–	0.0086 (0.0049,0.0152)	–
**38**	0.0378 (0.0197,0.0727)	0.0408 (0.0204,0.0816)	–	0.0069 (0.0034,0.0138)	–
**11B**	0.0381 (0.0191,0.0762)	0.0357 (0.017,0.0749)	–	0.0053 (0.0025,0.011)	–
**3**	0.0401 (0.0201,0.0802)	0.0571 (0.0272,0.1199)	–	0.0086 (0.0041,0.018)	–
**18C**	0.0417 (0.0199,0.0874)	0.039 (0.0175,0.0867)	–	0.0126 (0.0057,0.0281)	–
**11D**	0.0429 (0.0138,0.1329)	0.0429 (0.0138,0.1329)	–	0.0055 (0.0018,0.017)	–
**35F**	0.0476 (0.0179,0.1269)	0.0476 (0.0179,0.1269)	–	0.006 (0.0022,0.0159)	–

**NT**	0.0505 (0.0382,0.0669)	0.0466 (0.0339,0.064)	0.0714 (0.0396,0.129)	0.0093 (0.0068,0.0128)	0.0043 (0.0024,0.0077)
**28F**	0.0571 (0.0272,0.1199)	0.0571 (0.0272,0.1199)	–	0.009 (0.0043,0.019)	–
**8**	0.0595 (0.0248,0.143)	0.0595 (0.0248,0.143)	–	0.0089 (0.0037,0.0215)	–
**9N**	0.0625 (0.0298,0.1311)	0.0612 (0.0275,0.1363)	–	0.0057 (0.0026,0.0127)	–
**1**	0.0635 (0.0318,0.127)	0.0635 (0.0318,0.127)	–	0.0106 (0.0053,0.0212)	–
**41**	0.0714 (0.023,0.2215)	–	–	–	–
**19B**	0.0714 (0.0268,0.1903)	0.0714 (0.0268,0.1903)	–	0.0063 (0.0024,0.0167)	–
**47**	0.0714 (0.0268,0.1903)	0.0714 (0.023,0.2215)	–	0.0102 (0.0033,0.0316)	–
**47A**	0.0714 (0.0268,0.1903)	0.0714 (0.0268,0.1903)	–	0.0136 (0.0051,0.0363)	–
**5**	0.0714 (0.0268,0.1903)	0.0714 (0.0268,0.1903)	–	0.0053 (0.002,0.0141)	–
**10F**	0.0714 (0.0297,0.1716)	0.0714 (0.0297,0.1716)	–	0.0069 (0.0029,0.0167)	–
**16A**	0.0714 (0.0297,0.1716)	0.0714 (0.0297,0.1716)	–	0.0038 (0.0016,0.009)	–
**36**	0.0714 (0.0321,0.159)	0.0714 (0.0321,0.159)	–	0.0126 (0.0057,0.0281)	–
**19C**	0.0714 (0.0396,0.129)	0.0714 (0.0396,0.129)	–	0.0047 (0.0026,0.0085)	–
**47F**	0.0779 (0.0251,0.2416)	0.0779 (0.0251,0.2416)	–	0.007 (0.0022,0.0216)	–

Cells marked by "–" designate serotypes where no or very few data were available for analysis.

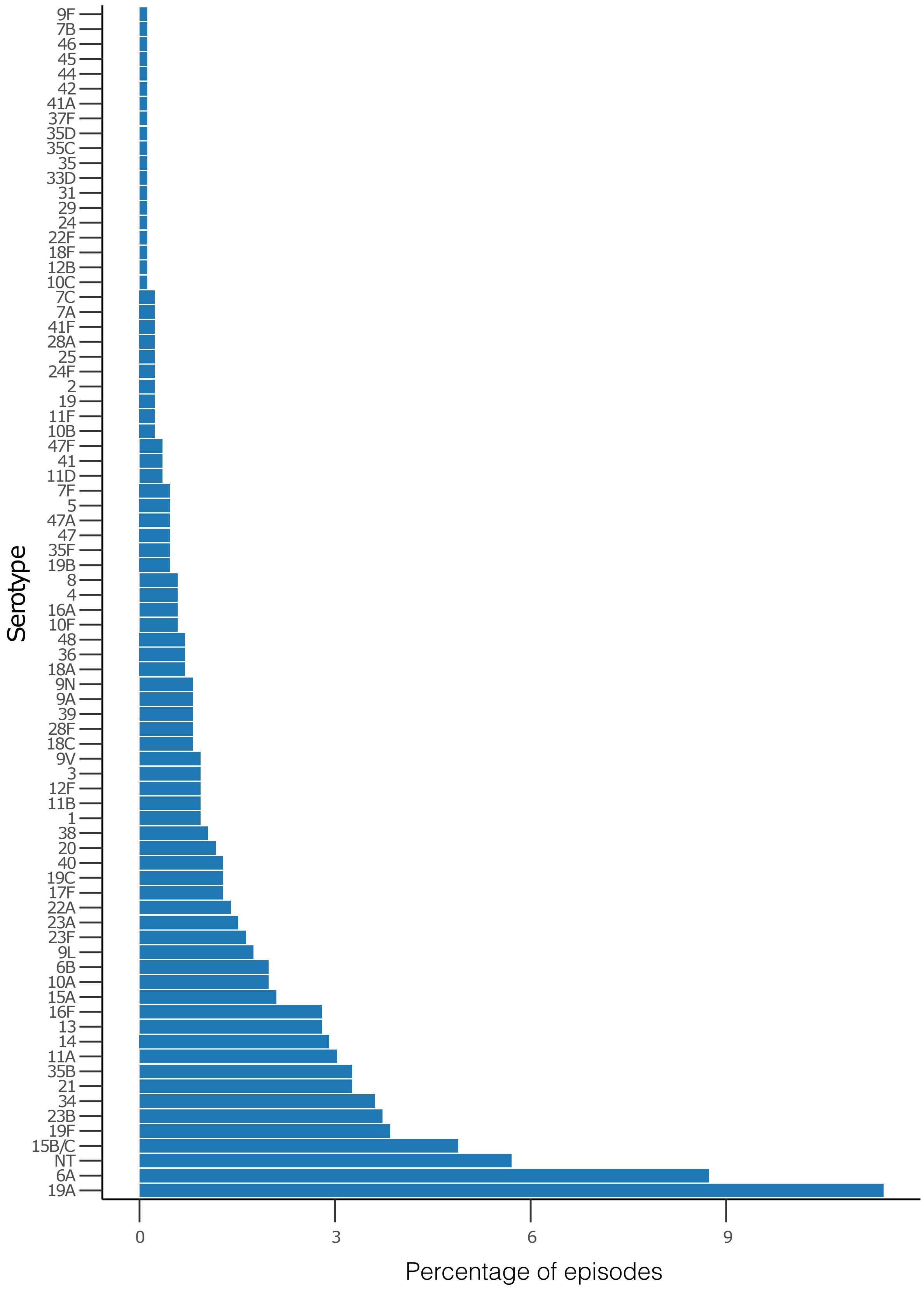

Supplementary Figure 1. Proportion of colonisation episodes associated with each serotype.

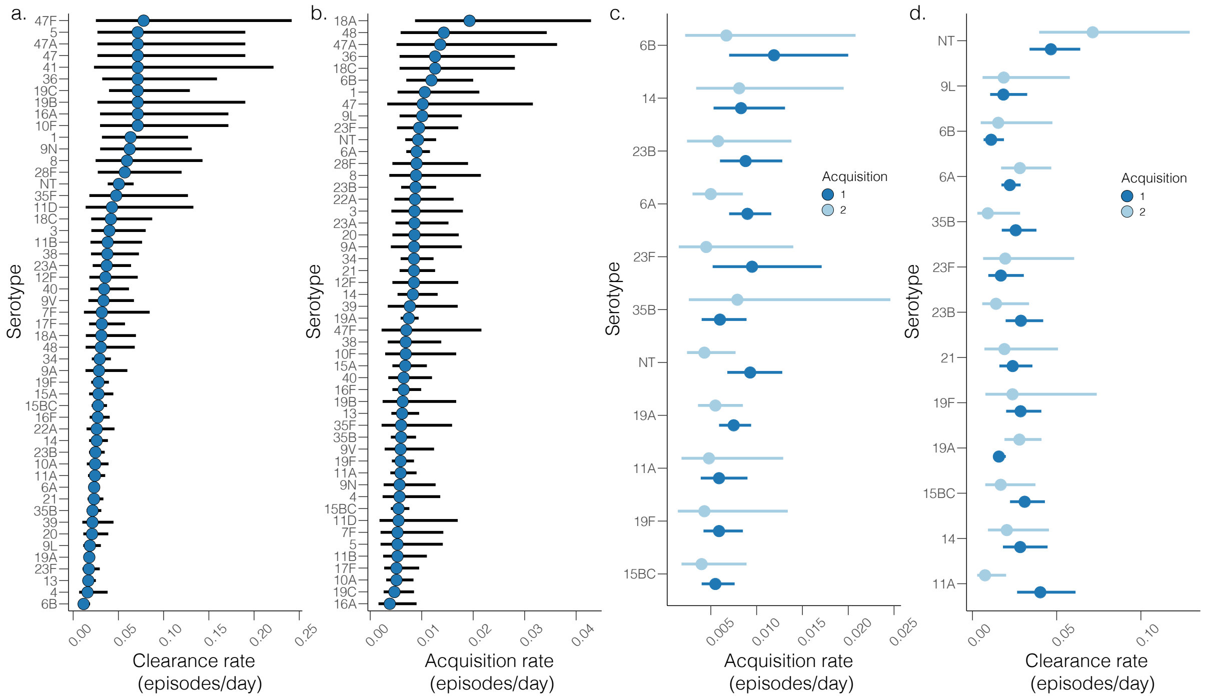

Supplementary Figure 2. Clearance and acquisition rates of pneumococcal serotypes in Gambian infants. Graph showing **(a)** clearance rate, **(b)** initial acquisition rate of serotypes, **(b)** acquisition rate of the second serotype, **(c)** rates of acquisition of a serotype for the first and second episode and **(d)** clearance rates of the first and second episode of a serotype.

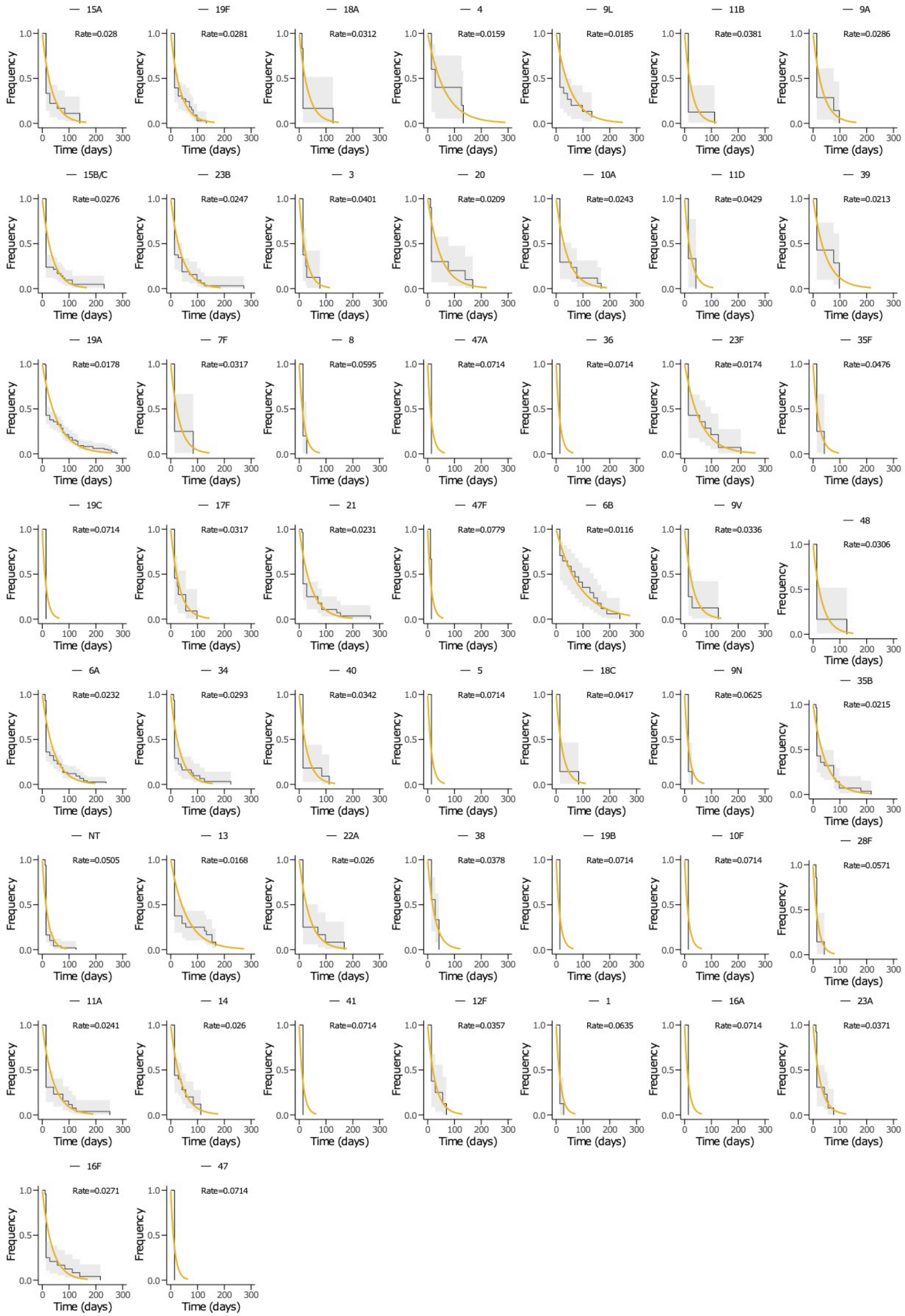

15A
Rate=0.028
19F
Rate=0.0281
18A
Rate=0.0312
4
Rate=0.0159
9L
Rate=0.0185
11B
Rate=0.0381
9A
Rate=0.0286
15B/C
Rate=0.0276
23B
Rate=0.0247
3
Rate=0.0401
20
Rate=0.0209
10A
Rate=0.0243
11D
Rate=0.0429
39
Rate=0.0213
19A
Rate=0.0178
7F
Rate=0.0317
8
Rate=0.0595
47A
Rate=0.0714
36
Rate=0.0714
23F
Rate=0.0174
35F
Rate=0.0476
19C
Rate=0.0714
17F
Rate=0.0317
21
Rate=0.0231
47F
Rate=0.0779
6B
Rate=0.0116
9V
Rate=0.0336
48
Rate=0.0306
6A
Rate=0.0232
34
Rate=0.0293
40
Rate=0.0342
5
Rate=0.0714
18C
Rate=0.0417
9N
Rate=0.0625
35B
Rate=0.0215
NT
Rate=0.0505
13
Rate=0.0168
22A
Rate=0.026
38
Rate=0.0378
19B
Rate=0.0714
10F
Rate=0.0714
28F
Rate=0.0571
11A
Rate=0.0241
14
Rate=0.026
41
Rate=0.0714
12F
Rate=0.0357
1
Rate=0.0635
16A
Rate=0.0714
23A
Rate=0.0371
16F
Rate=0.0271
47
Rate=0.0714
Frequency
Time (days)

Supplementary Figure 3. The Kaplan-Meier survival curves and exponential fit to the carriage duration data of each serotype. The plots show the overall duration from acquisition to clearance of each serotypes regardless of acquisition time. The black curve represents the Kaplan-Meier estimates while the yellow line is the fitted survival curve. The rate parameter in the exponential model is shown at the top of each plot represents the mean clearance rate whose inverse equates to mean the carriage duration.

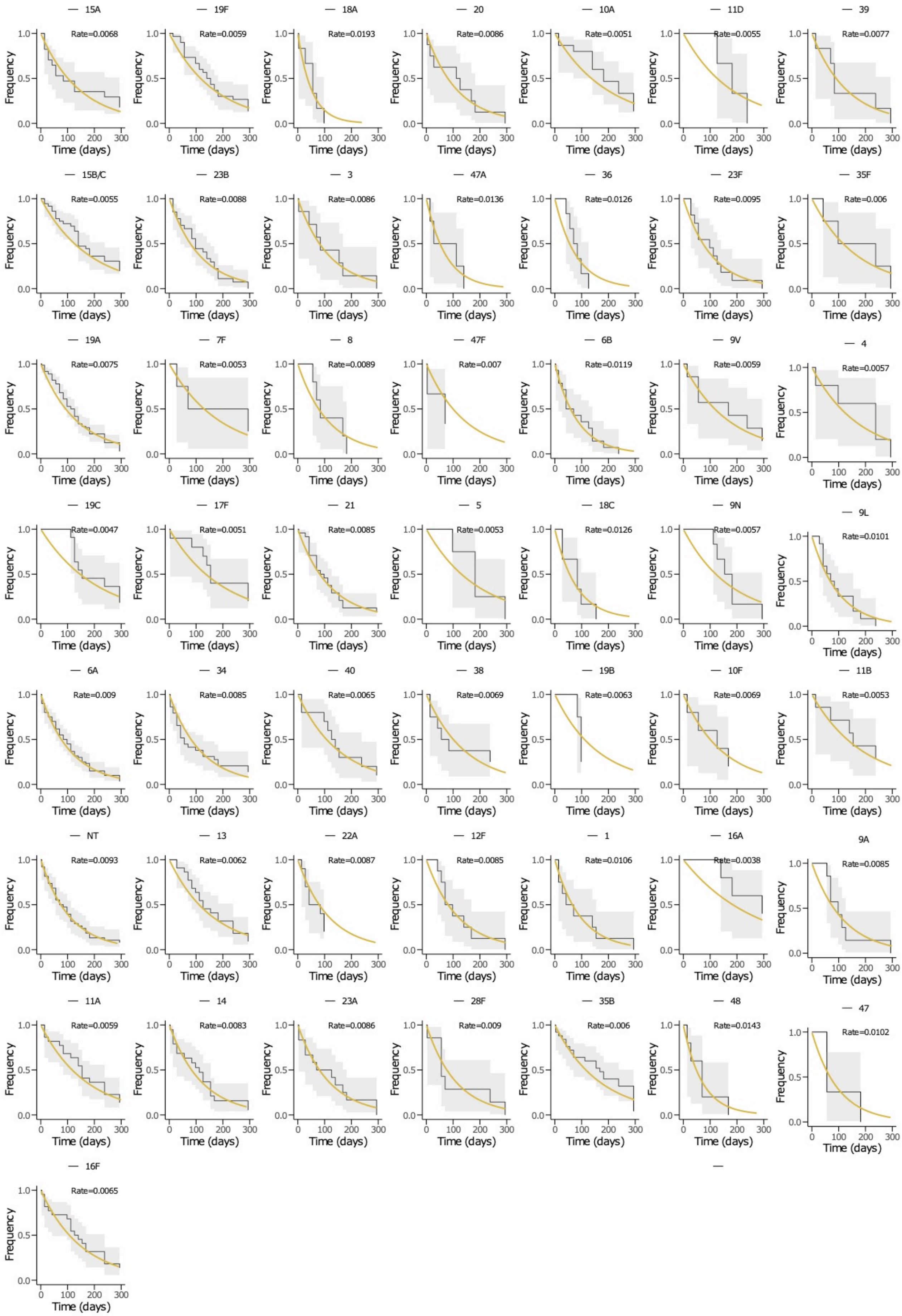
— 15A
Rate=0.0068
— 19F
Rate=0.0059
— 18A
Rate=0.0193
— 20
Rate=0.0086
— 10A
Rate=0.0051
— 11D
Rate=0.0055
— 39
Rate=0.0077
— 15B/C
Rate=0.0055
— 23B
Rate=0.0088
— 3
Rate=0.0086
— 47A
Rate=0.0136
— 36
Rate=0.0126
— 23F
Rate=0.0095
— 35F
Rate=0.006
— 19A
Rate=0.0075
— 7F
Rate=0.0053
— 8
Rate=0.0089
— 47F
Rate=0.007
— 6B
Rate=0.0119
— 9V
Rate=0.0059
— 4
Rate=0.0057
— 19C
Rate=0.0047
— 17F
Rate=0.0051
— 21
Rate=0.0085
— 5
Rate=0.0053
— 18C
Rate=0.0126
— 9N
Rate=0.0057
— 9L
Rate=0.0101
— 6A
Rate=0.009
— 34
Rate=0.0085
— 40
Rate=0.0065
— 38
Rate=0.0069
— 19B
Rate=0.0063
— 10F
Rate=0.0069
— 11B
Rate=0.0053
— NT
Rate=0.0093
— 13
Rate=0.0062
— 22A
Rate=0.0087
— 12F
Rate=0.0085
— 1
Rate=0.0106
— 16A
Rate=0.0038
9A
Rate=0.0085
— 11A
Rate=0.0059
— 14
Rate=0.0083
— 23A
Rate=0.0086
— 28F
Rate=0.009
— 35B
Rate=0.006
— 48
Rate=0.0143
— 47
Rate=0.0102
— 16F
Rate=0.0065
Frequency
Time (days)
0.0
0.5
1.0
0
100
200
300

Supplementary Figure 4. The Kaplan-Meier survival curves and exponential fit to the time to first acquisition data of each serotype. The plots show duration from birth of an infant to first acquisition of each serotype. The black curve represents the Kaplan-Meier estimates while the yellow line is the fitted survival curve. The rate parameter in the exponential model is shown at the top of each plot represents the mean acquisition rate whose inverse equates to mean the time to first acquisition of each serotype.

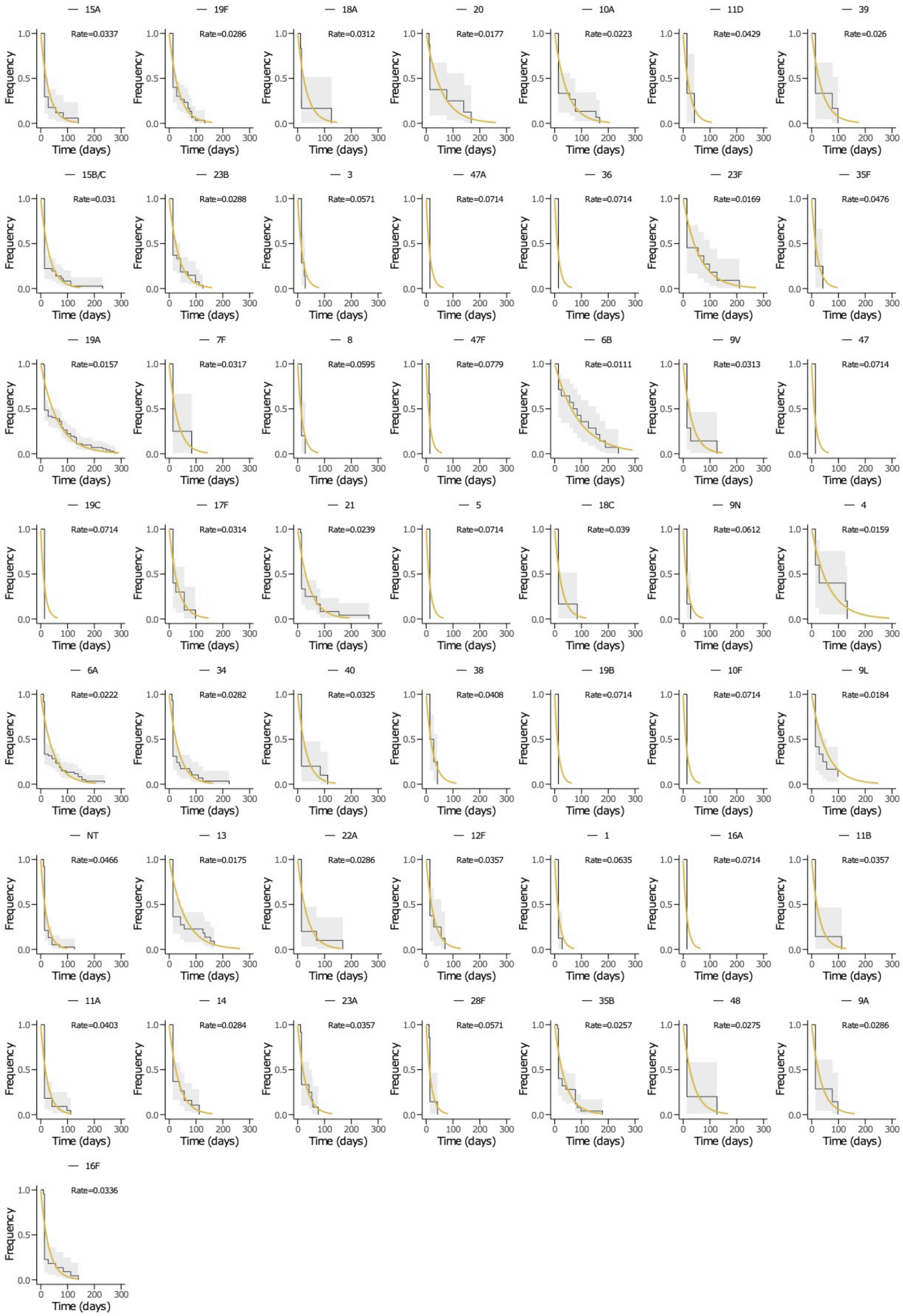
15A
Rate=0.0337
19F
Rate=0.0286
18A
Rate=0.0312
20
Rate=0.0177
10A
Rate=0.0223
11D
Rate=0.0429
39
Rate=0.026
15B/C
Rate=0.031
23B
Rate=0.0288
3
Rate=0.0571
47A
Rate=0.0714
36
Rate=0.0714
23F
Rate=0.0169
35F
Rate=0.0476
19A
Rate=0.0157
7F
Rate=0.0317
8
Rate=0.0595
47F
Rate=0.0779
6B
Rate=0.0111
9V
Rate=0.0313
47
Rate=0.0714
19C
Rate=0.0714
17F
Rate=0.0314
21
Rate=0.0239
5
Rate=0.0714
18C
Rate=0.039
9N
Rate=0.0612
4
Rate=0.0159
6A
Rate=0.0222
34
Rate=0.0282
40
Rate=0.0325
38
Rate=0.0408
19B
Rate=0.0714
10F
Rate=0.0714
9L
Rate=0.0184
NT
Rate=0.0466
13
Rate=0.0175
22A
Rate=0.0286
12F
Rate=0.0357
1
Rate=0.0635
16A
Rate=0.0714
11B
Rate=0.0357
11A
Rate=0.0403
14
Rate=0.0284
23A
Rate=0.0357
28F
Rate=0.0571
35B
Rate=0.0257
48
Rate=0.0275
9A
Rate=0.0286
16F
Rate=0.0336
Frequency
Time (days)

Supplementary Figure 5. The Kaplan-Meier survival curves and exponential fit to the carriage duration data after first acquisition for each serotype. The plots show duration from first acquisition until clearance of each serotype. The black curve represents the Kaplan-Meier estimates while the yellow line is the fitted survival curve. The rate parameter in the exponential model is shown at the top of each plot represents the mean clearance rate after first episode whose inverse equates to mean the time to acquisition of each serotype.

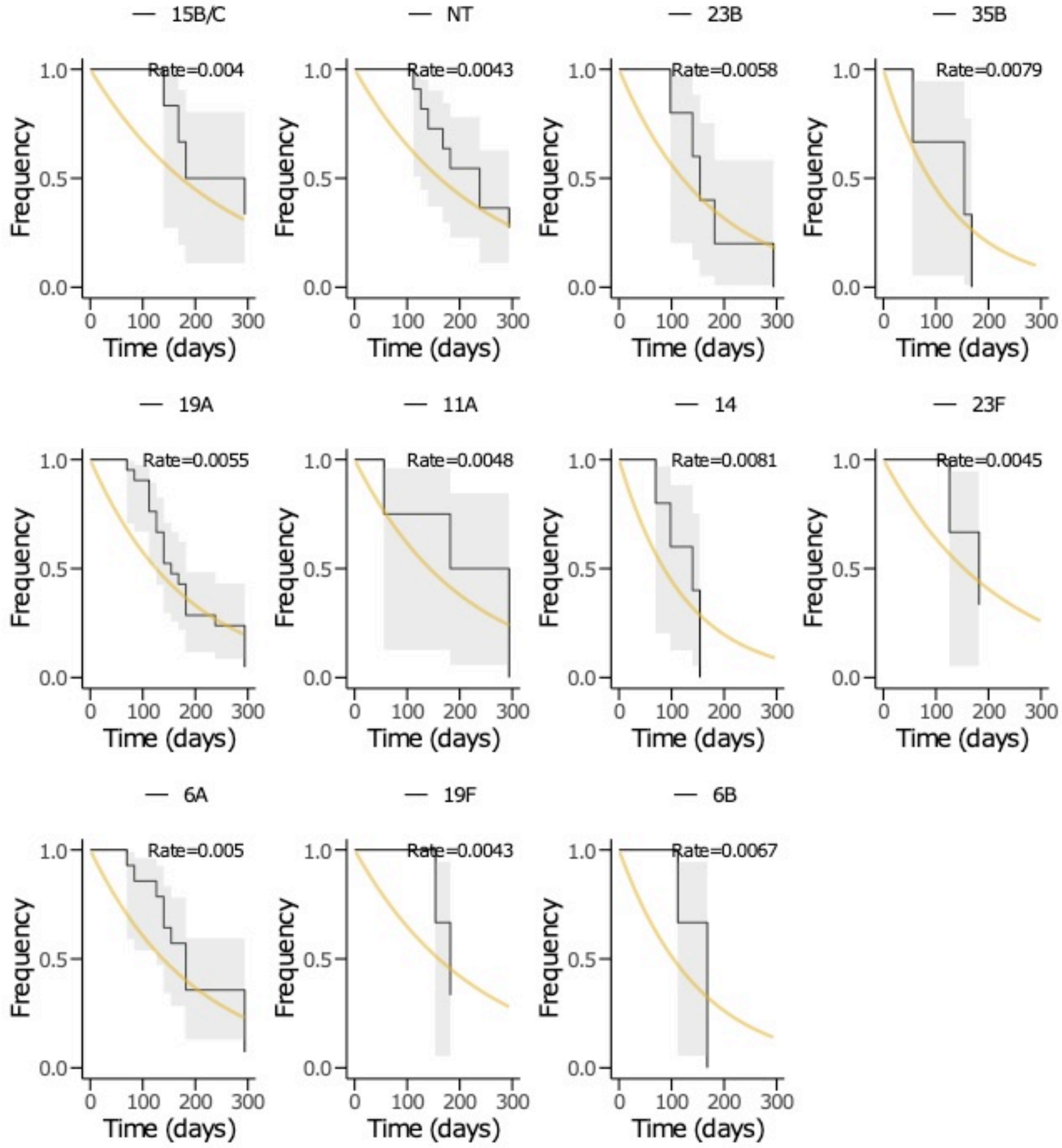

Supplementary Figure 6. The Kaplan-Meier survival curves and exponential fit to the time to second acquisition or reacquisition data for each serotype. The plots show duration from birth of an infant until second acquisition of each serotype. The black curve represents the Kaplan-Meier estimates while the yellow line is the fitted survival curve. The rate parameter in the exponential model is shown at the top of each plot represents the mean reacquisition rate after whose inverse equates to mean the time to reacquisition of each serotype.

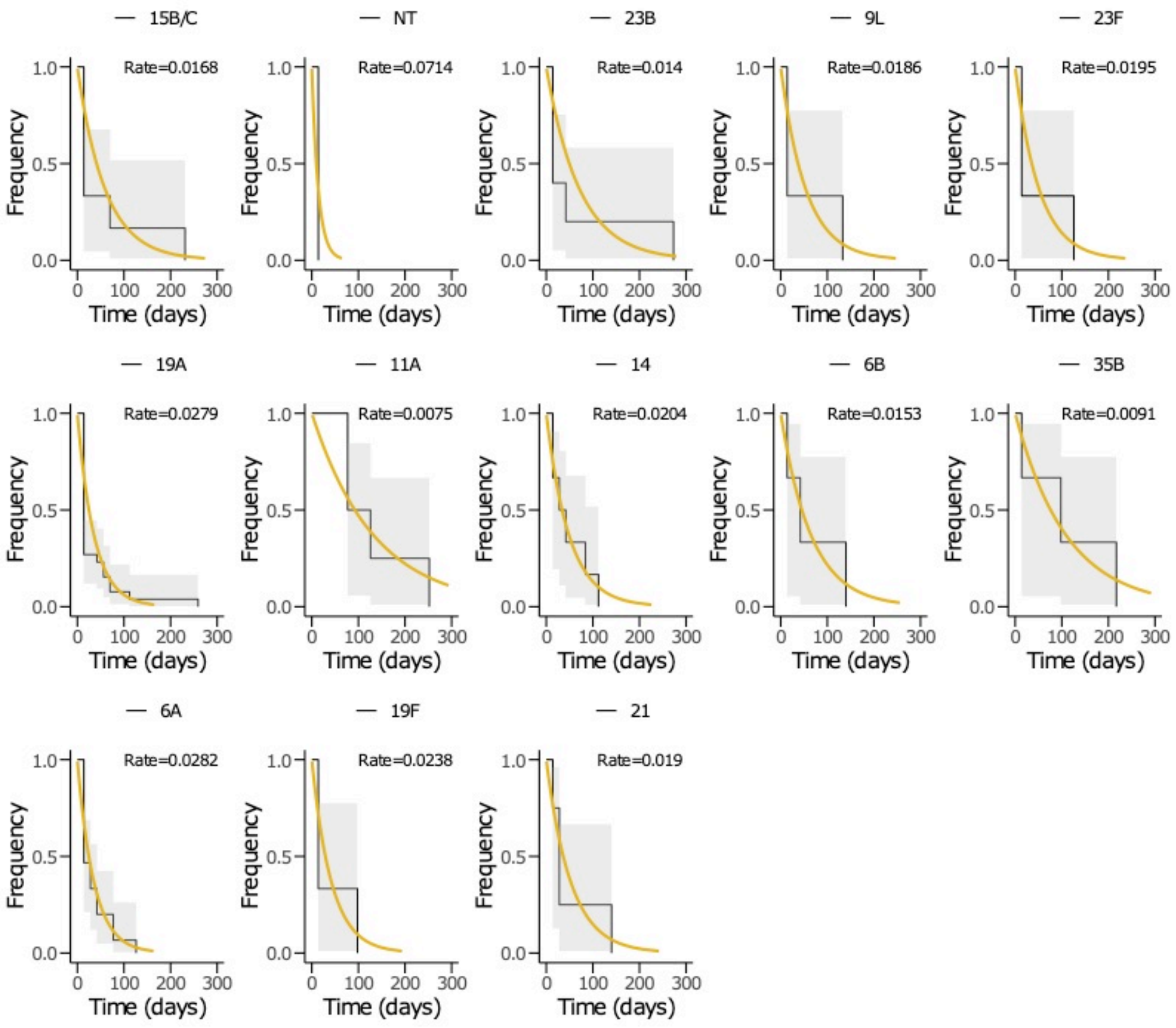

Supplementary Figure 7. The Kaplan-Meier survival curves and exponential fit to the carriage duration data after second acquisition or reacquisition for each serotype. The plots show duration from first acquisition until clearance of each serotype. The black curve represents the Kaplan-Meier estimates while the yellow line is the fitted survival curve. The rate parameter in the exponential model is shown at the top of each plot represents the mean clearance rate after first episode whose inverse equates to mean the time to acquisition of each serotype.

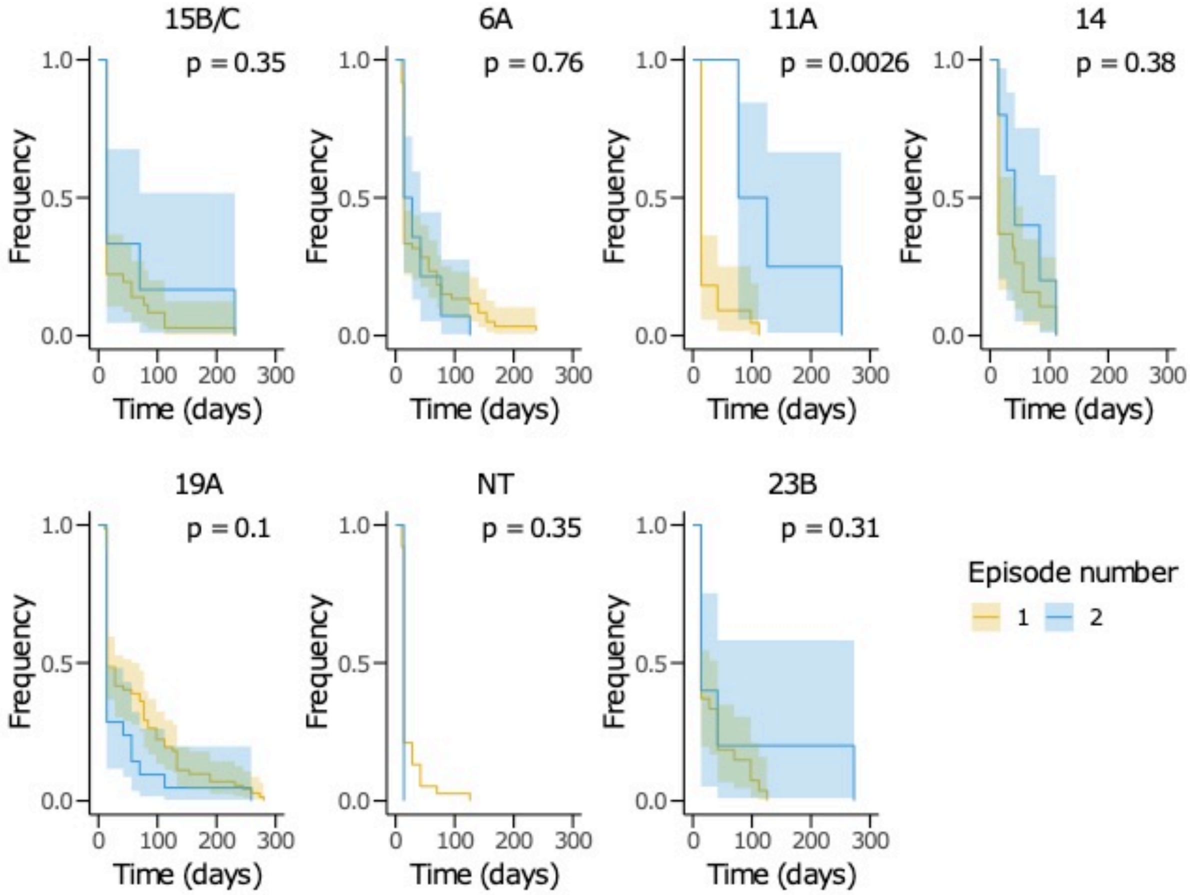

Supplementary Figure 8. The Kaplan-Meier survival curves comparing the carriage duration of the first episode and second episode of serotypes acquired multiple times. The *P*-values were calculated using the log-rank test.

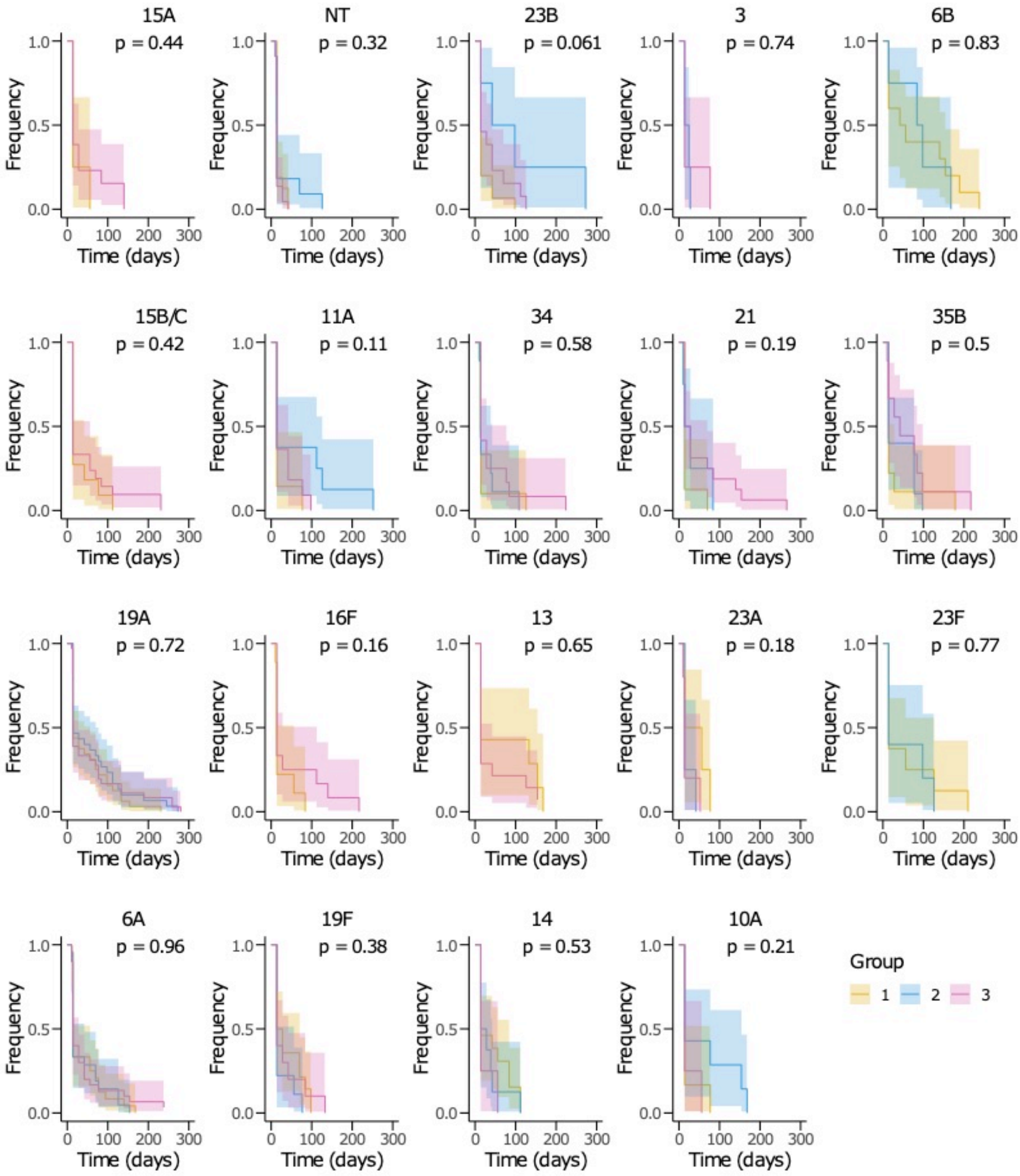

Supplementary Figure 9. The Kaplan-Meier survival curves comparing the carriage duration for each serotype in different communities. The infants in group 1 were from PCV-unexposed (unvaccinated) communities and received PCV7 after 6 months. The infants in group 2 were also from PCV-unexposed (unvaccinated) communities where the infants received PCV7 at 2, 3 and 4 months. The infants from group 3 originated from PCV-exposed (vaccinated) communities and they received PCV7 at 2, 3 and 4 months. The *P*-values were calculated using the log-rank test. Only serotypes found in both communities in >3 colonisation episodes are shown in the figure.

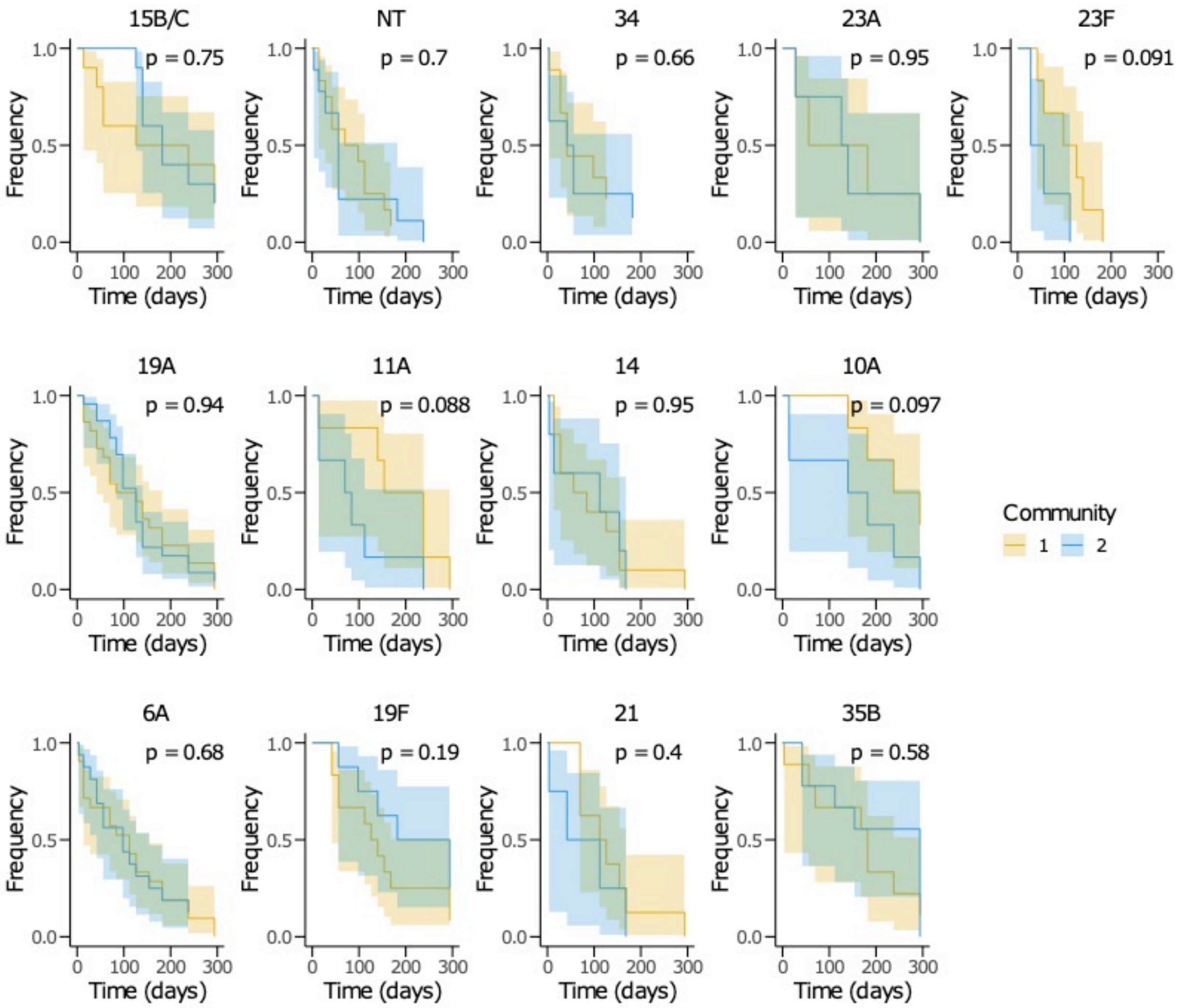

Supplementary Figure 10. The Kaplan-Meier survival curves comparing the time to acquisition for each serotype in different communities. The infants in community 1 were from PCV-unexposed (unvaccinated) villages and received PCV7 after 6 months. The infants in community 2 were also from PCV-unexposed (unvaccinated) villages where the infants received PCV7 at 2, 3 and 4 months. The infants from community 3 originated from PCV-exposed (vaccinated) villages and they received PCV7 at 2, 3 and 4 months. The *P*-values were calculated using the log-rank test. Only serotypes found in both communities in >3 colonisation episodes are shown in the figure.
